# Sex and gender in digital mental health: why the data are not neutral

**DOI:** 10.4069/whn.2026.08.03

**Published:** 2026-09-29

**Authors:** Chul-Hyun Cho

**Affiliations:** Departments of Psychiatry, Biomedical Informatics, and Medical Education, Korea University College of Medicine, Seoul, Korea

**Keywords:** Artificial intelligence, Health equity, Machine learning, Mental health, Sex factors

## Abstract

Digital tools, including smartphone sensors, wearable devices, and conversational artificial intelligence, have moved mental health assessment beyond the clinic and into everyday life, offering the possibility of earlier detection and broader access. However, these benefits are not distributed equally between women and men. Drawing on recent evidence, this review traces sex and gender across four layers of the digital mental health pipeline: who reaches these tools, how algorithms interpret the data, how effectively interventions perform, and how raw sensor signals are understood. At each layer, biases that may appear to be technical details instead reflect sex- and gender-related patterns. Women are more willing to try digital mental health services but are less likely to complete them, largely because of time scarcity rather than low motivation. Diagnostic algorithms trained on male-skewed data underperform for women, sometimes markedly. The same sensor signal, such as a shrinking movement radius or a decline in heart-rate variability, can carry different meanings depending on the user’s body and circumstances. These disadvantages can accumulate, leaving women doubly underserved. Yet the same evidence indicates that when sex and gender are incorporated into design, digital tools can reduce gaps rather than widen them. Sex and gender are not nuisance variables to be controlled away; they are parameters for precision care.

## Introduction

For most of its history, psychiatry has relied on a narrow slice of information. A patient recognizes that something is wrong, waits for an appointment, travels to a clinic, and spends perhaps 30 minutes describing the past few weeks. That account is retrospective and selective: the worst moments may be forgotten, and the days immediately before the visit can crowd out earlier experiences. Mood disorders, the severity of which fluctuates from day to day, are especially poorly captured by a single clinical snapshot.

Digital technology has begun to loosen that constraint. Smartphones and wearable devices now record behavior and physiology continuously and unobtrusively, turning the phone into an instrument for digital phenotyping and pairing it with ecological momentary assessment, so that experience can be captured as it occurs rather than reconstructed weeks later [1]. Conversational agents add another layer by offering support at any hour [2]. Together, these tools promise earlier detection, wider reach, and a fuller picture of a person’s life than any single clinic visit can provide.

Because sensors measure physical quantities, their data can appear neutral. They are not. Artificial intelligence learns from data that already carry the imprint of a field in which women have long been underrepresented; models can then reproduce and even intensify those patterns while appearing objective [3]. Decades of biomedical research point in the opposite direction: analyzing sex and gender deliberately, rather than ignoring them, produces better and more generalizable science [4]. Digital mental health has not yet fully absorbed that lesson.

This review follows sex and gender across four layers of the digital mental health pipeline: who reaches these tools, how algorithms interpret the data they generate, how well interventions work, and how raw sensor signals are read. The argument is cumulative: disadvantages introduced at one layer are rarely corrected downstream and are more often amplified (Figure 1). However, the same evidence also points to a way forward. When digital tools are designed with sex and gender in mind, those that currently fail women can instead serve a broader range of users more equitably.

## Sex, gender, and the data gap

The discussion must begin by distinguishing two ideas that are often conflated. Sex refers to biology, including chromosomes, anatomy, and hormones, and leaves a physical signature on sensor data. Vocal-cord length produces a higher baseline pitch in most women’s voices, with greater natural variation in frequency and amplitude; heart-rate variability shifts across the menstrual cycle; hormonal rhythms shape sleep architecture; and body composition affects how an accelerometer registers movement [5,6]. These patterns are not noise. They are signals that differ by sex.

Gender, by contrast, is social. It shapes whether a person feels able to seek help despite stigma, which services they are willing to accept, and how much uninterrupted time they can devote to those services. Men who endorse traditional ideas of masculinity may interpret logging a mood or opening a mental health app as a sign of weakness and leave early; women more often approach the same tools as a way to manage emotion and seek connection and therefore stay longer [7]. When German respondents chose between hypothetical services, women weighted whether the provider was human, whereas men weighted cost and waiting time, reflecting two different ways of valuing the same technology [8].

The framework of gendered innovations holds that incorporating sex and gender analysis from the outset makes research more rigorous and useful [4]. Its emphasis on intersectionality is especially relevant here because gender combines with income, age, and culture to produce very different outcomes. Older women in the United States readily adopted telehealth during the pandemic, extending skills first learned to stay in touch with family [9], whereas women in rural Bangladesh often could not access a consultation without a male relative’s permission or device [10]. The same gendered position can be an asset in one setting and a barrier in another.

This is the data gap. If the people, voices, and movement patterns used to train an algorithm are disproportionately male, the model learns a male default and treats female patterns as deviations. A single headline value for overall accuracy hides this problem; a more informative check is to report how performance varies by sex. The simplest available tool for this purpose is the disparate-impact ratio, defined as one group’s performance divided by the other’s, with values of approximately 0.8 to 1.2 generally considered acceptable [3].

## The access paradox

The first paradox appears at the point of entry into care. Women are consistently more willing than men to consider digital mental health services, yet across many datasets they are less likely to complete them [7,8]. Sign-up rates are similar; the divergence emerges after enrollment, shifting the question from motivation to what these tools require once a person begins using them.

What these tools require above all is time, and time is not distributed equally. Women remain the primary providers of unpaid care and domestic labor, so a program that asks for a fixed block of attention each day competes directly with someone else’s needs: a child’s homework, a parent’s phone call, or an evening meal [7]. For clinicians, the key point is that this is a structural constraint rather than an individual preference, and it should be treated as part of the design specification. Five-minute micro-sessions that are asynchronous and interruptible fit a caregiver’s life in a way that a fixed 30-minute block does not.

The pattern appears across very different settings. In Germany, the issue is the architecture of user preferences; among older adults in the United States, it is social motivation [9]; and in Bangladesh, it is what some authors call digital patriarchy, in which existing power imbalances are carried into digital access and intensified, leaving women with lower telehealth use and markedly higher rates of stopping medication [10]. A meta-analysis of engagement across 117 trials confirms that retention is shaped by modifiable design and contextual factors rather than by fixed user traits [11]. Geography and age vary, but the sex and gender axis runs through all these settings.

## Bias in the algorithm

Once data enter a model, bias takes new forms. The clearest demonstration comes from a counterfactual design. Rickman [12] used 617 real long-term-care records, changed only the pronouns and gendered terms while leaving every clinical fact unchanged, and then fed both versions to the same large language model under identical instructions. Because the medical content did not change, any difference between the two summaries could only reflect the patient’s apparent sex. Google’s Gemma model (Google LLC, Mountain View, CA, USA) summarized the same case differently: the male-coded record retained objective language about cognitive decline and fracture risk, whereas the female-coded version softened the same facts into emotional sensitivity and anxiety. This pattern reflects diagnostic overshadowing, the long-criticized practice of recasting women’s physical complaints as emotional ones, now learned and reproduced by software. Meta’s Llama 3 (Meta Platforms, Inc., Menlo Park, CA, USA) did not show the same gap. This contrast suggests that the bias reflected one model’s training choices rather than an unavoidable feature of the technology, and that model selection is itself a clinical decision.

The failure can also operate in two directions at once. When machine-learning models were trained to detect anorexia from social media posts, the false-negative rate for women was many times higher than for men: the models dismissed genuine warning signs as ordinary interest in dieting or appearance [13]. For men, the problem was the mirror image. Stigma limits men’s disclosure of eating-disorder symptoms, leaving too few cases for models to learn from, so detection failed through data scarcity [14]. Broader reviews of text-based prediction reach the same conclusion: without attention to who is represented in the training data, models inherit the blind spots of the corpus [15].

Sensor-based assessment shows how large the resulting gap can be. Using mobile-phone data in a random-forest classifier, Park et al. [6] reported an accuracy of 87.16% for men but only 71.31% for women, a gap of 15.85 percentage points, with a disparate-impact ratio of 0.68, well outside the fairness range. Because the sample was small, this estimate is best understood as proof of principle rather than as a population-level estimate. The encouraging finding is that a single preprocessing step to rebalance the data narrowed the gap to 1.66 percentage points, with only a slight reduction in overall accuracy, from 78.57% to 77.12%. Fairness, in other words, can be engineered. The broader governance literature is beginning to formalize this approach by classifying model risks according to how they affect different users and contexts and by treating sex as a variable that cuts across several risk categories at once [16,17].

## When sex-aware design closes gaps

The corrective evidence is equally important. In a randomized trial of internet-delivered interpersonal psychotherapy and cognitive behavioral therapy for depression, female sex was one of the strongest predictors of both greater symptom reduction and higher compliance [18]. A component network meta-analysis that pooled 76 trials and more than 18,000 patients went further by identifying which treatment ingredients mattered: behavioral activation added meaningful benefit, relaxation could worsen outcomes, and combining human support with the automated program sharply reduced dropout [19]. The practical implication is that a single one-size-fits-all program may lose men, whereas a hybrid pathway with a human-in-the-loop can bring them back.

Good design can also erase a starting disadvantage. In a smartphone cognitive behavioral therapy trial for adolescent insomnia, girls entered with worse sleep than boys, yet after six weeks both groups improved comparably, with no significant interaction between gender and time [20]. Equity in this study was not assumed; it was observed as an outcome of the design. A Korean trial points in the same direction within a pregnancy-specific life stage: a 4-week self-guided mindfulness app for pregnant women significantly reduced anxiety and improved emotional well-being and maternal-fetal attachment, although depression and stress did not change [21]. This pattern underscores the value of reporting both positive and null outcomes.

One caveat tempers this optimism. Pooled comparisons still favor in-person therapy on average: across more than 100 studies, face-to-face cognitive behavioral therapy outperformed digital cognitive behavioral therapy in symptom reduction and adherence [22]. Many women nonetheless choose digital care because it avoids the travel, exposure, and time costs associated with in-person treatment. For them, the relevant comparison is not effect size in the abstract but what is realistically attainable. Dropout data reinforce the same point. In a large insomnia trial, overall dropout rates for men and women were almost identical, and demographic factors did not significantly moderate efficacy; however, the reasons for dropout differed [23]. Men tended to leave because they judged the program ineffective or too slow, whereas women tended to leave because they ran out of time or missed human connection. One number concealed two different problems, each requiring its own fix.

## The sensor is not neutral

The most subtle bias lies in the raw signal, where the same number can mean different things in different bodies and lives. A shrinking radius of movement is a classic marker of social withdrawal and, by extension, depression, but for a woman it may simply reflect a caregiving loop among home, school, and local errands, with no change in mood at all [10]. A high vocal pitch with greater variability may be read as instability only when judged against a male baseline; for many women, it is the normal starting point [6]. A decline in heart-rate variability may appear to indicate autonomic dysregulation, yet during the luteal phase of the menstrual cycle it is ordinary physiology, repeating every month for roughly half the population [5]. An algorithm that does not know where a woman is in her cycle can therefore misread normal variation as an anxiety alert. Table 1 presents several of these signals alongside their default and sex-aware interpretations.

The remedies are straightforward. Sensor pipelines can normalize data within sex before training, treating biological baselines as explicit variables rather than errors to be averaged away [5]. They can account for differences in device hardware and for the fact that device preference is itself gendered, preventing a dataset skewed toward one platform from quietly becoming skewed toward one sex. They can also record the social context in which a signal is generated, including occupation, household structure, and caregiving role, because an ambiguous reading of mobility or sleep timing cannot be interpreted correctly without that context. Context is part of the signal, not a distraction from it [1].

## Governance and the path forward

Good intentions and technically sound methods are insufficient unless development and reimbursement incentives require equity to be measured. The World Health Organization’s 2024 guidance on large multimodal models in health emphasizes inclusive design with underrepresented groups and auditing after deployment, rather than only before release [24]. In digital mental health, these principles can be operationalized as a staged pipeline: rebalancing data before training, constraining the model for fairness during training, and adjusting decision thresholds afterward, so that equity is checked at every stage rather than bolted on at the end [3]. Table 2 distills these steps into a seven-step checklist for product teams.

Regulation has not yet caught up. Digital therapeutics are currently cleared without any requirement for sex-stratified subgroup analysis, which means that bias often becomes visible only after a product reaches the market. A reasonable and specific regulatory request would be to make such analysis mandatory through minimum per-sex sample sizes, effect-size comparisons across sexes in pivotal trials, and post-market reporting that triggers action when a gap exceeds a defined threshold. Korea’s regulator has begun reviewing digital therapeutics but does not yet require sex-subgroup analysis [25]; under the European Union’s 2024 framework, mental-health artificial intelligence is still treated as medium-risk, a classification that the World Health Organization has argued should be raised. Payers also have a role: if a tool works for one sex but not the other, reimbursement should reflect that difference.

These layers are not independent, and that is the crux of the problem. A woman may be kept from finishing a program by time poverty and, if she does engage, may then have her data misread by a model tuned to male patterns; in that scenario, an access barrier and an algorithmic barrier stack into a double disadvantage [26]. As one analysis argues, the democratization of health care is a means rather than an end; the real test is whether it produces health justice [27]. A compounding problem requires a coordinated response across all four layers, rather than a patch applied to any single layer in isolation.

One blind spot deserves explicit attention. Almost all of this evidence sorts people into male and female categories, leaving non-binary and transgender users—groups at higher risk of poor mental health—largely unstudied and often unable even to register accurately on platforms that offer only two options [28]. A genuinely inclusive field will need to measure gender as more than a binary and report the experiences of minority groups separately, rather than allowing them to disappear into the average. Even within the binary, usability work with older adults suggests that groups often assumed to struggle with technology can use these tools well when they receive brief training and support, which is another reason not to design for an imagined average user [29].

## Conclusion

The central message of this review is easy to state but difficult to act on. Across access, algorithms, interventions, and sensor data, sex and gender shape outcomes at every step. Ignoring them does not make a system neutral; it makes the system biased in favor of the users whom the data already represent. The same sensor, model, and program that underserve women today can serve a broader range of users tomorrow, but only if sex and gender are treated as design parameters rather than afterthoughts. For clinicians and researchers in women’s health, the practical stance is to stop treating sex and gender as obstacles to be controlled away and to start using them as variables that make care more precise. For nurses and nursing researchers, the implications are concrete. Nurses often introduce and sustain these tools with patients, placing them in a position to notice when a device misreads a woman’s data and to advocate for sex- and gender-sensitive design. Nursing researchers can contribute by reporting outcomes separately by sex and gender, including women across life stages and gender-diverse people in validation studies, and testing whether tools fit the caregiving and time constraints that shape women’s engagement. Whether a digital tool serves its users well depends less on its technical sophistication than on whom it is built to include; by that measure, digital mental health still has work to do.

## Figures and Tables

**Figure 1.**
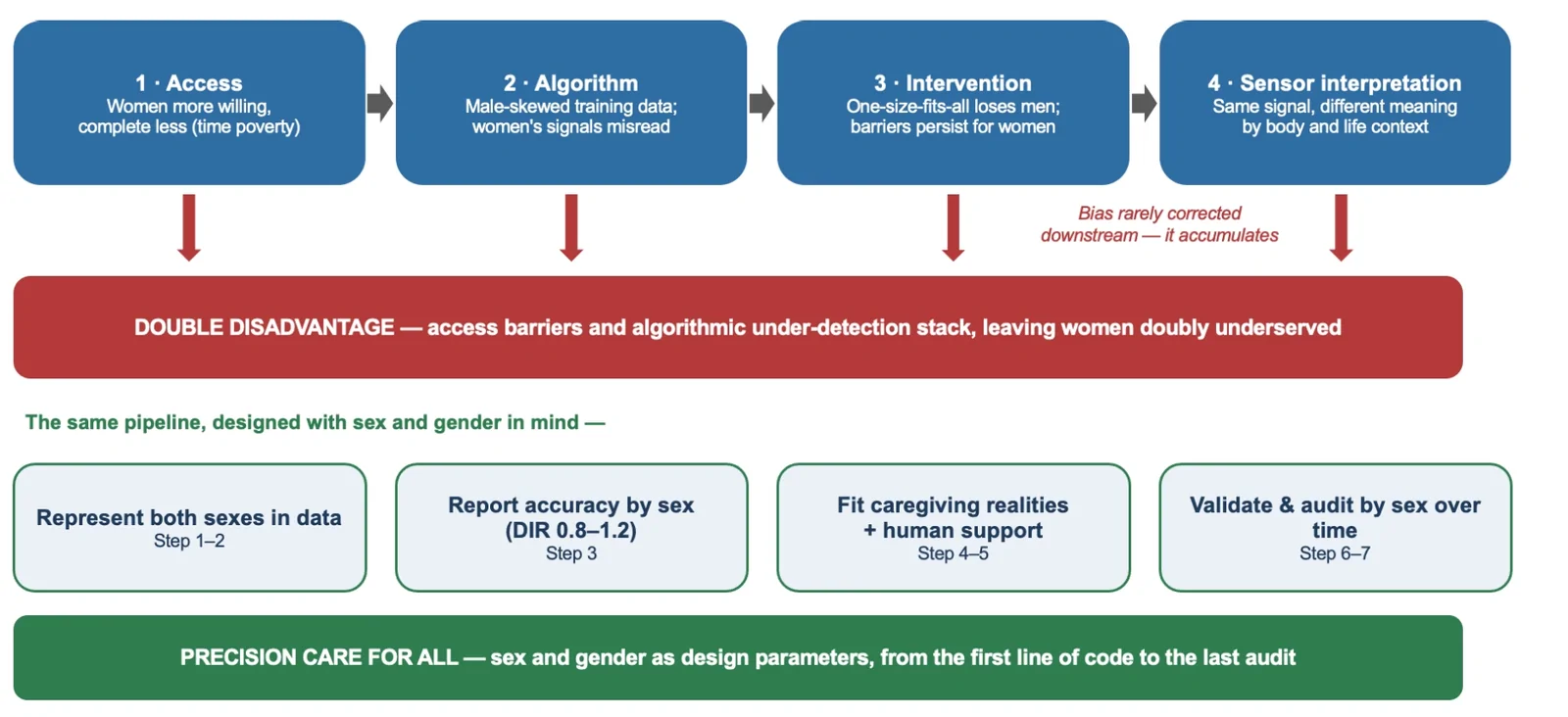
Compounding sex and gender bias across the digital mental health pipeline. Bias introduced at each layer—access, algorithm, intervention, and sensor interpretation—is rarely corrected downstream and is more often amplified, producing a cumulative “double disadvantage” for women. Sex- and gender-aware design applied at every layer can convert the same pipeline into a route toward precision care. DIR: Disparate-impact ratio.

**Table 1.** Default versus sex-aware interpretations of common digital signals

Signal	Default interpretation	Sex-aware interpretation	Design response
GPS: shrinking radius of gyration	Social withdrawal → depression	May reflect a caregiving loop rather than low mood [10]	Add location entropy and trip purpose, not distance alone
Voice: high fundamental frequency with jitter	Emotional instability → anxiety	May represent a normal female vocal baseline [6]	Set acoustic baselines within sex
Heart-rate variability: luteal-phase decline	Autonomic dysregulation → anxiety	May reflect ordinary menstrual-cycle variation [5]	Stratify by sex and add cycle-phase metadata
Activity: low daytime movement	Psychomotor retardation	May reflect care work or safety constraints on mobility [10]	Capture social-context metadata

The examples are illustrative and context-dependent rather than fixed clinical rules; identical sensor values can support opposite clinical readings depending on the user’s sex and life context. GPS: Global positioning system.

**Table 2.** Seven-step checklist for sex-aware digital mental health design

Step	Question to ask
1. Data representation: sex and gender	Are sex and gender categories adequately represented in the training data?
2. Baseline calibration: sex	Are sensor baselines normalized within sex before modeling?
3. Algorithmic error: sex and gender	Is accuracy reported by sex and gender, with a disparate-impact ratio of 0.8–1.2?
4. Interface and content: gender	Does the design avoid gender stereotypes and fit caregiving realities, such as micro-sessions and asynchronous flow?
5. Human support: gender	Is a human-in-the-loop option available for users who disengage without it?
6. Clinical validation: sex and gender	Has effectiveness been tested and reported separately for each sex and gender category?
7. Post-deployment monitoring: sex and gender	Is performance audited over time for bias drift, including among gender-diverse users and beyond binary categories where possible?

Developed based on World Health Organization guidance on large multimodal models [24] and the bias-mitigation pipeline of Timmons et al. [3].
